# Evolutionary and natural history of the turtle frog, *Myobatrachus gouldii*, a bizarre myobatrachid frog in the southwestern Australian biodiversity hotspot

**DOI:** 10.1371/journal.pone.0173348

**Published:** 2017-03-15

**Authors:** Samantha Vertucci, Mitzy Pepper, Danielle L. Edwards, J. Dale Roberts, Nicola Mitchell, J. Scott Keogh

**Affiliations:** 1 Division of Evolution, Ecology and Genetics, Research School of Biology, The Australian National University, Canberra, Australia; 2 School of Natural Sciences, University of California, Merced, CA, United States of America; 3 Centre of Excellence in Natural Resource Management, The University of Western Australia, Albany, Western Australia; 4 School of Animal Biology, The University of Western Australia, Crawley, Australia; State Museum of Natural History, GERMANY

## Abstract

Southwest Australia (SWA) is a global biodiversity hotspot and a centre of diversity and endemism for the Australo-Papuan myobatrachid frogs. *Myobatrachus gouldii* (the turtle frog) has a highly derived morphology associated with its forward burrowing behaviour, largely subterranean habit, and unusual mode of reproduction. Its sister genera *Metacrinia* and *Arenophryne* have restricted distributions in Western Australia with significant phylogeographic structure, leading to the recent description of a new species in the latter. In contrast, *Myobatrachus* is distributed widely throughout SWA over multiple climatic zones, but little is known of its population structure, geographic variation in morphology, or reproduction. We generated molecular and morphological data to test for genetic and morphological variation, and to assess whether substrate specialisation in this species may have led to phylogeographic structuring similar to that of other plant and animal taxa in SWA. We assembled sequence data for one mitochondrial and four nuclear DNA loci (3628 base pairs) for 42 turtle frogs sampled throughout their range. Likelihood phylogenetic analyses revealed shallow phylogeographic structure in the mtDNA locus (up to 3.3% genetic distance) and little variation in three of the four nDNA loci. The mtDNA haplotype network suggests five geographically allopatric groups, with no shared haplotypes between regions. These geographic patterns are congruent with several other SWA species, with genetic groups restricted to major hydrological divisions, the Swan Coastal Plain, and the Darling Scarp. The geographically structured genetic groups showed no evidence of significant morphological differentiation (242 individuals), and there was little sexual size dimorphism, but subtle differences in reproductive traits suggest more opportunistic breeding in lower rainfall zones. Call data were compared to sister genera *Metacrinia* and *Arenophryne* and found to be highly conservative across the three genera. Like many taxa in SWA, topographic variation and Plio-Pleistocene arid fluctuations likely were historic drivers of diversification in *M*. *gouldii*.

## Introduction

South Western Australia (SWA) is an internationally recognised biodiversity hotspot [1, 2]. With a temperate Mediterranean climate, the region covers over 300 000 km^2^ and is a centre of endemism for both plants (79% endemic to SWA) and animals, particularly amphibians (80% endemic to SWA) [3, 2]. The exceptionally high biodiversity of the southwest has provoked much interest as the region is topographically subdued and has experienced no recent large-scale vicariance events, such as glaciation or tectonic activity, that are often associated with such high levels of speciation [4].

**Fig 1:**
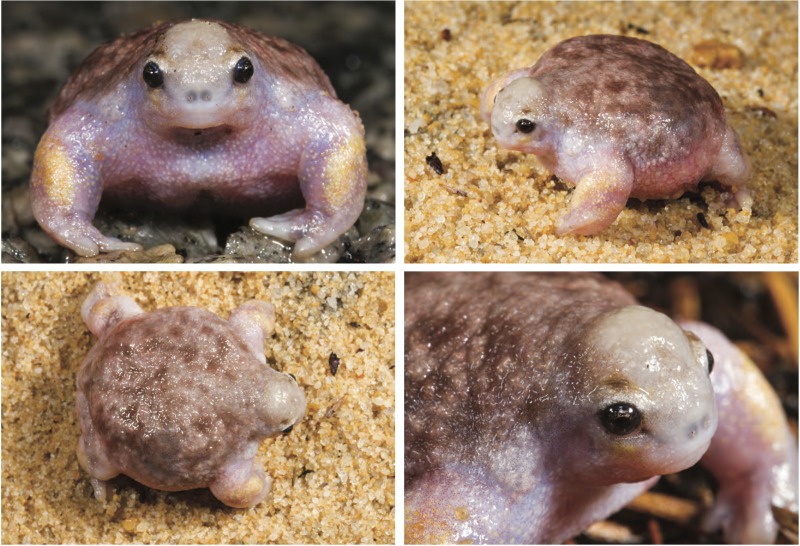
*Myobatrachus gouldii* live, *in situ*, Kalbarri National Park, WA. Photo credit: Stephen Zozaya.

Increasing aridity following the Miocene and subsequent arid pulses throughout the Pleistocene are thought to have played a significant role in the diversification of many species in the southwest [5–7], with *in situ* speciation characteristic of many endemic frogs and reptiles [8]. Prior to the mid-Tertiary, palynological, fossil and geological evidence indicates higher rainfall and a warmer climate than currently present across SWA, with a high proportion of subtropical rainforest plant species [9]. Aridification of SWA began in the mid to late Tertiary after separation of Australia from Antarctica [10]. A distinct increase in aridity occurred at the Plio-Pleistocene border [11] followed by repeated, deeply arid pulses during glacial cycles of the Pleistocene. These periods saw the arid and semi-arid zones expand further, with the most recent increase in aridity occurring during the last glacial maximum. Another factor thought to drive speciation and present day floral and faunal patterns in SWA has been the formation of lateritic soils in the Oligocene and/or Miocene, and the continued weathering of the landscape resulting in the edaphic (soil) diversity in the southwest today [12, 4]. While the southwest is generally topographically subdued, the Darling Scarp (see Fig 1), an escarpment dividing the Yilgarn craton from the Swan Coastal Plain, has been identified as a barrier to gene flow in some taxa [13, 14]. Although there are few phylogeographic studies in SWA, emerging patterns suggest the SWA flora and fauna comprises a mixture of ancient and recently diverged lineages, with much of this diversity undescribed [3, 6–8, 12–18].

The Australo-Papuan myobatrachid frogs are a large radiation that includes 21 genera and 129 species, and this group is particularly diverse in SWA [19, 20]. One clade of myobatrachids that evolved *in situ* in SWA comprises four highly distinctive species: *Metacrinia nichollsi*, *Arenophryne rotunda*, *A*. *xiphorhyncha* and *Myobatrachus gouldii*. All species have direct-developing (endotrophic) eggs, are fossorial, do not hop, and have a reduced 4^th^ finger. *Metacrinia* more closely resembles a typical myobatrachid anuran, while the *Arenophryne* species and *Myobatrachus* display highly derived morphologies associated with their primarily subterranean lifestyles, including a fusiform shape, reduced head and eyes [21–23], and short muscular arms adapted for burrowing forward through sandy soils (most other Australian fossorial frogs use their hind feet to burrow backwards [24]). These unique morphological adaptations are most pronounced in the monotypic genus *Myobatrachus*. Also known as the turtle frog, *M*. *gouldii* is a “distinctive globular frog” [25] that feeds largely on termites [26] (Fig 1). As in *Arenophryne*, *Myobatrachus* is a terrestrial breeder, eggs can be laid more than a metre below ground, and surface behaviour is usually only observed after rain when frogs emerge to forage or for courtship [27]. Since its taxonomic description [28], only a handful of studies have dealt specifically with the turtle frog. These have identified aspects of feeding habits [26], physiology [22, 29] and reproduction [27, 30–34]. However, the fossorial habit of this species has resulted in an incomplete account of its natural history within the published literature.

**Fig 2:**
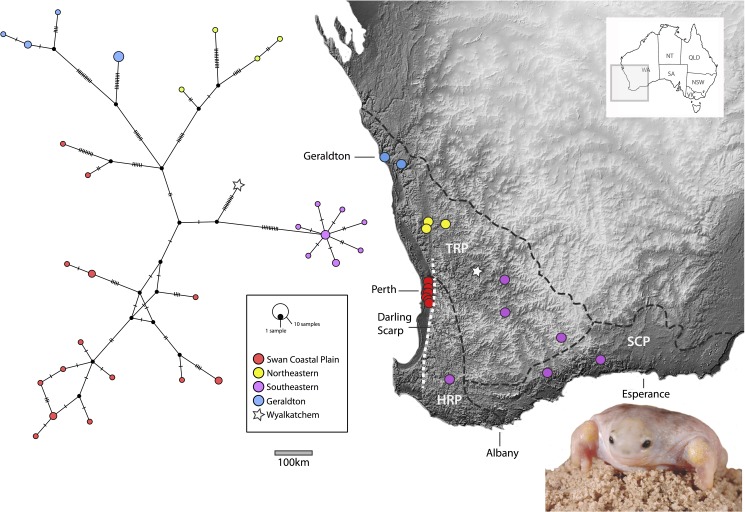
Haplotype network of 42 *Myobatrachus gouldii* samples based on the combined ND2 and RPL35 data, with mutations shown as cross bars. The distribution of samples throughout south-western Australia are overlain onto a digital elevation model image (Shuttle Radar Topography Mission) where light grey equates to areas of high elevation, and dark grey equates to areas of low elevation. Black dashed line on the map represents the rainfall provinces after Hopper & Goia [38] (TRP: Transitional Rainfall Province, HRP: High Rainfall Province, SCP: South Coast Province), while the white dotted line refers to the location of the Darling Scarp. (image of *M*. *gouldii* from Yanchep National Park courtesy of M. Anstis).

Phylogenetic relationships between *Myobatrachus* and its sister taxa are well established [17, 35–37], yet no phylogenetic hypothesis of relationships among *M*. *gouldii* populations has been available. Population level phylogeographic structuring was recently uncovered within *Metacrinia* [37], and population and species-level fragmentation in *Arenophryne* [17], leading to the description of the formerly cryptic *A*. *xiphorhyncha* [23]. Both these taxa have restricted distributions within SWA, and each is confined to a single climatic zone. In contrast, *Myobatrachus gouldii* is widely distributed throughout SWA, from north of Geraldton to east of Esperance, across the Transitional Rainfall Zone and the Southeast Coastal Province, as well as the outskirts of the High Rainfall Province (Fig 2). The distribution of the turtle frog over multiple climatic zones, its dependence on deep sandy substrate, as well as its subterranean lifestyle and expected low vagility suggests that *Myobatrachus* could show phylogeographic patterns similar to other plants and animals in the region. We set out to test this hypothesis with molecular and morphological data and we also provide detailed information on call structure and reproduction.

## Materials and methods

### Molecular data and analyses

Tissue samples were obtained from the frozen tissue collections of the Western Australian Museum, the South Australian Museum, and the private collection of NM (UWA Animal Ethics Committee: 06-100-586; DPaW (then DEC) scientific license: SF 005585). All *M*. *gouldii* tissue samples available in Australia at the time of our study were included, giving a total of 42 samples (38 to 42 were sequenced for each gene) (Table 1). The geographic coverage of these samples against the known distribution of *M*. *gouldii* is reasonable but a number of collecting gaps remain (see S1 Fig for distribution records and Fig 2 for the distribution of genotyped individuals). Additional tissue collection to fill in gaps in remote areas was not practical because the frogs only appear sporadically on the soil surface after rains, which are unpredictable across the more arid regions of its range. We also quantified morphology for a much larger group of animals (see below and S1 Fig for the distribution of morphotyped individuals). *Metacrinia nicholsii*, *Arenophryne rotunda* and *A*. *xiphorhyncha* were included due to their close relationship with *M*. *gouldii* and as a comparison of intra-specific genetic distances [36, 37].

**Table 1:** Locality information for all *Myobatrachus gouldii* specimens sequenced in this study. Each sample was given a laboratory identification number (Lab ID). Museum number refers to the number of the corresponding voucher specimen at the Western Australian Museum (WAM) or South Australian Museum (SAM). The locality information specifies the nearest named location as provided by the museum.

Lab ID	Museum	Latitude	Longitude	Locality
MGX01	WAMR103745	-33.8833	118.9333	10km NNE Jerramungup
MGX02	WAMR115076	-31.9333	115.7667	Bold Park
MGX03	WAMR115077	-31.9333	115.7667	Bold Park
MGX04	WAMR115078	-31.9333	115.7667	Bold Park
MGX05	WAMR115103	-32.0833	115.8833	Ken Hearst Park
MGX06	WAMR115133	-32.0833	115.8833	Ken Hearst Park
MGX07	WAMR115975	-32.9333	119.15	20 Km Ne Newdegate
MGX08	WAMR116075	-28.65	114.6333	Spalding Park, Geraldton
MGX09	WAMR116076	-28.65	114.6333	Spalding Park, Geraldton
MGX10	WAMR116077	-28.65	114.6333	Spalding Park, Geraldton
MGX11	WAMR116216	-31.9	115.8333	Tuart Hill
MGX12	WAMR120300	-28.7333	115	Wicherina Reserve
MGX13	WAMR120305	-28.7333	115	Wicherina Dam
MGX14	WAMR120306	-28.7333	115	Wicherina Dam
MGX15	WAMR125665	-31.8158	115.7781	Hepburn Heights, Perth
MGX16	WAMR127623	-31.6667	115.75	Neerabup
MGX17	WAMR141704	-30.1447	115.7606	Watheroo National Park
MGX18	WAMR141705	-30.0325	115.8183	25km SW Coorow
MGX19	WAMR144078	-31.5119	117.7303	13km N Kellerberrin
MGX20	WAMR144079	-31.5119	117.7303	13km N Kellerberrin
MGX21	WAMR144139	-31.9666	115.8	Shenton Park
MGX22	WAMR144140	-31.9666	115.8	Shenton Park
MGX23	WAMR144141	-31.9666	115.8	Shenton Park
MGX24	WAMR144405	-32.0166	115.8333	Jandakot Airport
MGX25	WAMR144894	-30.05	116.2333	Marchagee Area
MGX26	WAMR144895	-30.05	116.2333	Marchagee Area
MGX27	WAMR146470	-32.3333	117.7333	12km W Corrigin
MGX28	WAMR146471	-32.3333	117.7333	12km W Corrigin
MGX29	WAMR156756	-33.5727	120.3078	Bandalup Hill
MGX30	WAMR156757	-33.5683	120.3197	Bandalup Hill
MGX31	WAMR156759	-33.5683	120.3197	Bandalup Hill
MGX33	WAMR154370	-31.2833	117.0333	Hindmarsh Nature Reserve
MGX36	WAMR116074	-28.65	114.6333	Spalding Park, Geraldton
MGX37	WAMR116415	-28.7333	115	Wicherina
MGX39	SAMAR40264	-33.95	120.1167	Hopetown
MGX40	WAMR116034	-33.9672	116.3256	Winnejup
MGX41	WAMR136390	-31.95	115.7667	Bold Park
MGX42	NM Collection	-31.566	115.8184	15km E Yanchep NP
MGX43	NM Collection	-31.566	115.8184	15km E Yanchep NP
MGX44	NM Collection	-31.566	115.8184	15km E Yanchep NP
MGX45	NM Collection	-31.566	115.8184	15km E Yanchep NP
MGX46	NM Collection	-31.566	115.8184	15km E Yanchep NP

We generated sequence data for five loci, including a 1200 base pair region of the mtDNA gene ND2 and associated tRNAs to facilitate comparison with detailed population and species level studies in the sister taxa *Metacrinia nicholsii* and *Arenophryne* species [17, 37]. ND2 has been used extensively in similar studies on other frogs, e.g. [39, 40]. Additionally, four nDNA genes were sequenced: a 480 base pair region of Pro-opiomelanocortin (POMC), a 600 base pair region of neurotrophin 3 (NTF3), a 700 base pair region of Brain-derived neurotrophic factor (BDNF), and a 380 base pair region of intron Ribosomal protein L3, intron 5 (RPL3int5). All four genes were selected because they also show variation at the inter-specific level [41–45]. *Spicospina flammocaerulea* was included as an outgroup, based on the phylogeny of Read *et al*. [36]. All new sequences from this study are deposited on Genbank (accession numbers KY084756-KY084832).

PCR and cycle sequencing methods are outlined in detail in S1 Text, and the primers used are detailed in Table 2. All sequences were run on an ABI 3100 auto-sequencer. Sequences were edited and assembled using Sequencher 3.0 (Genes Codes Corporation) and aligned by eye. Protein-coding regions were translated into amino acid sequences using the vertebrate mitochondrial and universal genetic codes, to check for stop codons, frame shifts and other signs of nuclear paralogs.

**Table 2:** The names, sequences and sources of the various primers used in this study. Primers were used for both PCR amplification and sequencing unless otherwise specified.

Gene	Primer Name	Sequence (5’-3’)	Source
POMC	POMC_F1	ATATGTCATGASCCAYTTYCGCTGGAA	Morgan pers comm
POMC	POMC_R1	GGCRTTYTTGAAWAGAGTCATTAGWGG	Morgan pers comm
BDNF	BDNF_Fmb	GACCATCCTTTTCCTKACTATGGTTATTTCATACTT	Morgan pers comm
BDNF	BDNF_Rmb	CTATCTTCCCCTTTTAATGGTCAGTGTACAAAC	Morgan pers comm
Ntf3	NTF3_F3	TCTTCCTTATCTTTGTGGCATCCACGCTA	Morgan pers comm
Ntf3	NTF3_R3	ACATTGRGAATTCCAGTGTTTGTCGTCA	Morgan pers comm
ND2	L4437	AAGCTTTCGGGGCCCATACC	Macey *et al*, 1998
ND2	L4882	CMACVTGRCAAAAAYTAGCCCC	Edwards, 2007 (modified from Melville *et al* 2004)
ND2	H5591_Ala	GTAATATAGAGTTTTACAGGC	Edwards, pers comm
ND2	MyoND1B	TTCCTATGAGTWCGWGCATCA	Edwards, pers comm
RPL	RPL35F	AAGAAGTCYCACCTCATGGAGAT	Pinho *et al* 2009
RPL	RPL36RA	AGTTTCTTTGTGTGCCAACGGCTAG	Pinho *et al* 2009
RPL	RPL36R	TTRCGKGGCAGTTTCTTTGTGTGCCA	Pinho *et al* 2009

Preliminary analyses of the individual nDNA loci revealed almost no variation in BDNF, POMC and NTF3 (see S2 Fig). The intron RPL3 was the most variable of the nDNA genes, with *M*. *gouldii* exhibiting 17 haplotypes. For final phylogenetic analyses we therefore chose to concatenate only ND2 and RPL3. Following the removal of ambiguously aligned nucleotide sites, the final ND2 dataset consisted of 1413 base-pairs (bp), and RPL3 consisted of 384 bp, totaling 1797 bp for the concatenated dataset. Combined data were partitioned by codon position for ND2 (excluding the tRNA which was treated as a single partition) and the non-coding intron was treated as a single partition. PartitionFinder [46] was used to establish the best partitioning strategy for the analysis, using linked branch lengths, RaxML model of evolution, and with the best model selected based on the Akaike Information Criterion (3 subset partitions; [ND2_tRNA, ND2_pos1] [ND2_pos2, RPL3] [ND2_pos3]). Phylogenetic analyses were conducted using maximum likelihood (ML) in RAxML-VI-HPC v7.0.4 [47]. The general time-reversible substitution model with gamma-distributed rates among sites (GTR + G) was implemented, with the best ML tree determined using 20 distinct randomized Maximum Parsimony (MP) starting trees. Bootstrap support was determined using 1000 replicates. A TCS haplotype network was also generated using the ND2 dataset and the PopART program [48].

### Morphological data and analyses

We collected extensive morphological data from 242 individuals (adults only, >15mm SVL) from across the known range of *M*. *gouldii* to assess morphological variation. Each animal was scored for 21 morphological characters (Table 3), which were adapted from a morphological study of the sister genus *Arenophyne* [23]. Snout-vent length (SVL) was measured to the nearest mm, inter-limb length (ILL), wrist width (WrW), arm width (AW), tibiofibular length (tibL) and shoulder width (ShW) to 0.1mm and the remainder to 0.01mm with digital callipers. All specimens were sexed by direct examination of the reproductive organs.

**Table 3:** Characters comprising the morphometric data set, modified from [23].

Character	Abbrev.	Explanation of Measurement
Snout-vent length	SVL	From tip of snout to posterior tip of urostyle
Inter-limb length	ILL	From axilla to groin
Head length	HL	From tip of snout to posterior edge of midpoint of tympanic fold
Head length 2	HL2	From tip of snout to angle of jaw
Head width	HW	Width of head at midpoint of tympanic fold
Eye-naris distance	EN	From anterior corner of eye to posterior edge of naris
Interorbital span	IO	Distance between anterior corners of eyes
Internarial span	IN	Distance between inner edges of nares
Eye length	EyeL	Anterior to posterior corners of eye
Mouth width	MthW	Distance between corners of the mouth.
Tympanic membrane width	TMW	(Anterior to posterior) Diameter of Tympanic membrane
Shoulder width	ShW	Distance between tips of pectoral girdles
Hand length	HandL	Tip of 3^rd^ finger to proximal edge of palmar tubercle
Finger length	FL	Length of 3^rd^ finger
Thumb length	TL	From tip of 1^st^ finger to the join between 1^st^ and 2^nd^ finger.
Wrist width	WrW	Width of wrist
Arm width	ArmW	Maximum width of forearm
Tibial length	TibL	Knee to heel (tibiofibular), leg in normal resting position where possible
Foot length	FootL	From tip of 4^th^ toe to distal edge of inner metatarsal tubercle
4^th^ Toe length	TL1	Length of 4^th^ toe
1^st^ Toe length	TL2	From tip of 1^st^ toe to join between 1^st^ and 2^nd^ toes

Exploratory morphological analyses showed little evidence of structure. Therefore, we used the genetic results (Fig 2) to assign specimens to three geographically definable regions as a means of testing for morphological differentiation between these groups: Geraldton region (N = 14), Swan Coastal Plain and northeastern region (N = 140) and southeastern region (N = 87). Principal Components Analysis (PCA) was used to examine the patterns of relationship among all 21 morphological characters (natural log transformed). PCA analyses were performed with the statistics software JMP 8.0. As not all specimens could be measured for all characters (due to occasional poor specimen preservation), we calculated standard principal components and imputation of missing values so that all 242 animals could be included. Analyses based on non-imputed data gave the same result (not shown). Plots of PC1 and PC2, and PC2 and PC3 were examined for evidence of morphological differentiation between the three haplotype groups. To test for sexual dimorphism we also conducted a series of analysis of covariance (ANCOVA) tests using snout-vent length, hand length, foot length or head width as the covariate, as appropriate for the individual variable. We also re-ran the PCA tests to visualize differences, if any, between males and females based on a combination of characters.

### Reproductive data and analyses

Where possible, testis size or the size of the largest follicle or egg were recorded to the nearest 0.1mm for all animals in the morphological dataset. Incidental observations such as oviduct characteristics in females were also noted as they are indicative of time since oviposition (NM unpublished data and [30]). Clutch sizes were not measured due to the extreme variation in follicle size in *M*. *gouldii* ovaries (e.g. <2.0–7.0 mm) and because very small follicles are difficult to count accurately [30].

Reproductive data were excluded from analyses if the collection date was unknown, which was true for 31 specimens collected before 1955. Data were then grouped by clade and sex (5 males and 8 females from the Geraldton clade, 35 males and 68 females from the Swan Coastal Plain/northeastern clade, and 35 males and 35 females for the southeastern clade. A gonadosomatic index (GSI; dimension of testis or follicle/SVLx100) was calculated for each individual and plotted against month of collection to identify any differences in reproductive seasonality among groups. If months of peak reproductive maturity could be identified, then data were filtered to include only this subset of months, and mean GSI were compared between clades for each sex.

### Call structure

**Fig 3:**
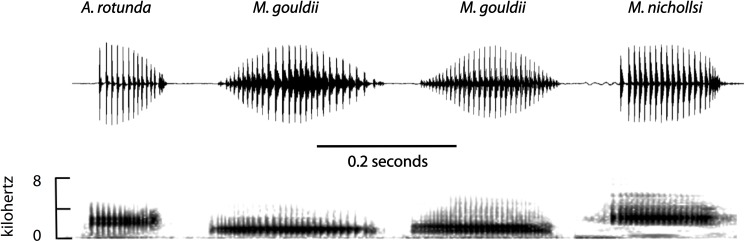
**Representative calls for *Arenophryne rotunda* from Shark Bay, *Myobatrachus gouldii* from (a) Yanchep and (b) Cullacabardee north of Perth, and *Metacrinia nichollsi* from Albany, Western Australia**. Bar represents 0.2 seconds.

We have illustrated calls of *Myobatrachhus gouldii* from two sites north of Perth, and, the closely related species *Arenophyrne rotunda* and *Metacrinia nichollsi* for comparison. Calls were recorded as follows: *Myobatrachus gouldii* call 1) Nov 3, 1985, tape # JDR 73, locality 31.81313°S, 115.93411°E, Jarrah's End Road, Yanchep, Beyer M88 microphone, call 2) Feb 10, 1980, tape # JDR 52, 31.50614°S, 115.67993°E, Park Street, Cullacabardee, AKG D190 microphone–both recorded on a Sony TC 510–2 reel to reel recorder. *Arenophryne rotunda*, Aug 8, 1980, tape # JDR 52, 26.39272°S, 113.31043°E, False Entrance, Shark Bay, Beyer M101 microphone, Sony TC 510–2 reel to reel recorder. *Metacrinia nichollsi*, Feb 27, 2015, digital File #1010, 35.02723°S, 117.89447°E, Mt Clarence, Albany, Marantz PMD620MKII digital recorder with inbuilt microphone. Oscillograms and sound spectrograms of calls (Fig 3) were made using Raven Pro, Version 1.4 (see http://www.birds.cornell.edu/brp/raven/ravenoverview.html) with playback for tape recordings made on recorders used to make original recordings. There are detailed descriptions of calls of *A*. *rotunda* and *M*. *gouldii* in Roberts [27, 31].

## Results

### Molecular data and analyses

The phylogeny based on our combined ND2 and RPL3 data exhibited phylogeographic structure within *M*. *gouldii*, with some well supported clades but with lower support for the deeper relationships among them. While we present the phylogeny in S3 Fig, due to the shallow nature of the ML topology, our results are more easily interpreted when viewed as a haplotype network. A total of 31 unique and phylogeographically structured mitochondrial haplotypes were identified, and while some individuals within a region had the same haplotype, there were no shared haplotypes between regions (Fig 2). The distributions of the main haplotype groups are shown in Fig 2. In the northern extreme of the range near Geraldton, Spalding Park individuals and Wicherina individuals each comprise well-supported haplotype groups (bs = 100 & 97, respectively). ML analyses identified a sister group relationship between these two groups, but with only moderate support (bs = 85). These two haplotype groups are geographically only 35km apart but have an uncorrected genetic distance of 1.7–1.9% in ND2. The individuals in the southeastern haplotype group were all collected east of the Darling Scarp, in the SWA wheatbelt region. This well supported clade (bs = 94) comprised a number of closely related haplotypes, which display a maximum uncorrected genetic distance of only 0.37% in ND2. A single individual from the central wheatbelt near Wyalkatchem (WAMR154370 from Hindmarsh Nature Reserve) had a unique haplotype 1.5%-2.9% divergent in ND2 from all other individuals. Individuals from the Swan Coastal Plain group (in and around Perth) and the northeastern group displayed the greatest diversity in haplotypes. Swan Coastal Plain animals were not monophyletic in the ML phylogeny but were distributed across two distinct haplotype groups (Fig 2). Individuals from Bold Park were found in each of these haplotype groups and differed by 1.3% in ND2. The northeastern haplotypes were monophyletic in the ML phylogeny and displayed up to 1.7% uncorrected ND2 genetic distance within the group. The maximum uncorrected genetic distance within *M*. *gouldii* was 3.3% between Wicherina and Swan Coastal Plain animals. The intron RPL3 was the most variable of the nDNA genes, with *M*. *gouldii* exhibiting 17 haplotypes. These haplotypes did not display phylogeographic structure, with the exception of Spalding Park animals, which were monophyletic (not shown).

### Morphological data and analyses

A total of 242 individuals from throughout the range of *M*. *gouldii* were included in the morphometric data set (specimen numbers in S2 Text). Sexually mature frogs varied in SVL between 26mm and 54mm (males) and 27mm and 63mm (females). Each specimen was assigned to one of the three main haplotype groups identified by analysis of the mtDNA genetic data (Fig 2 and S3 Fig) and corresponding to their geographic location (Fig 2). While animals from the Swan Coastal Plain and northeastern region did not form a monophyletic group with respect to the other clades in the mtDNA tree, the specimens from these regions were collapsed into a single group, as haplotypes from within the Swan Coastal Plain groups were sympatric and because morphological sample sizes were low for animals from the north-eastern part of the range.

**Fig 4:**
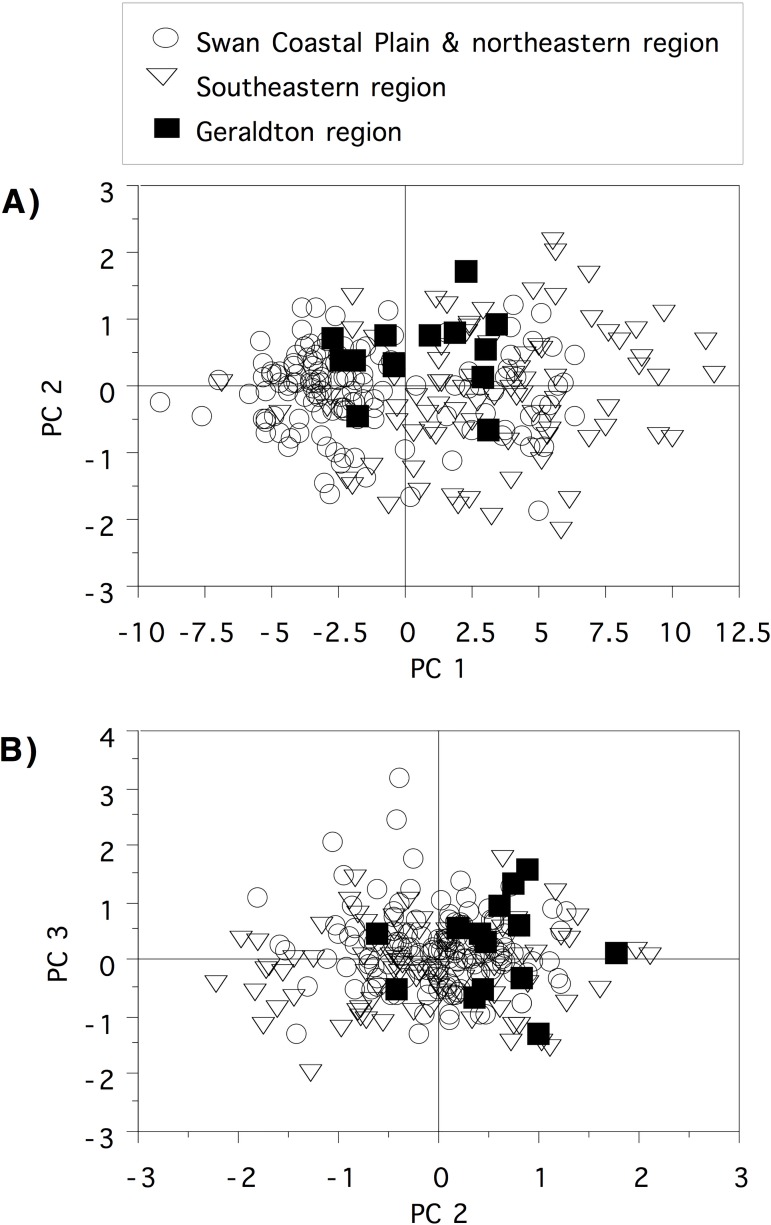
**Plots of the first and second (A) and second and third (B) Principle Components based on analysis of the morphological data set (see text for details) to show differences between the three primary hapotype groups / geographic regions**.

**Fig 5:**
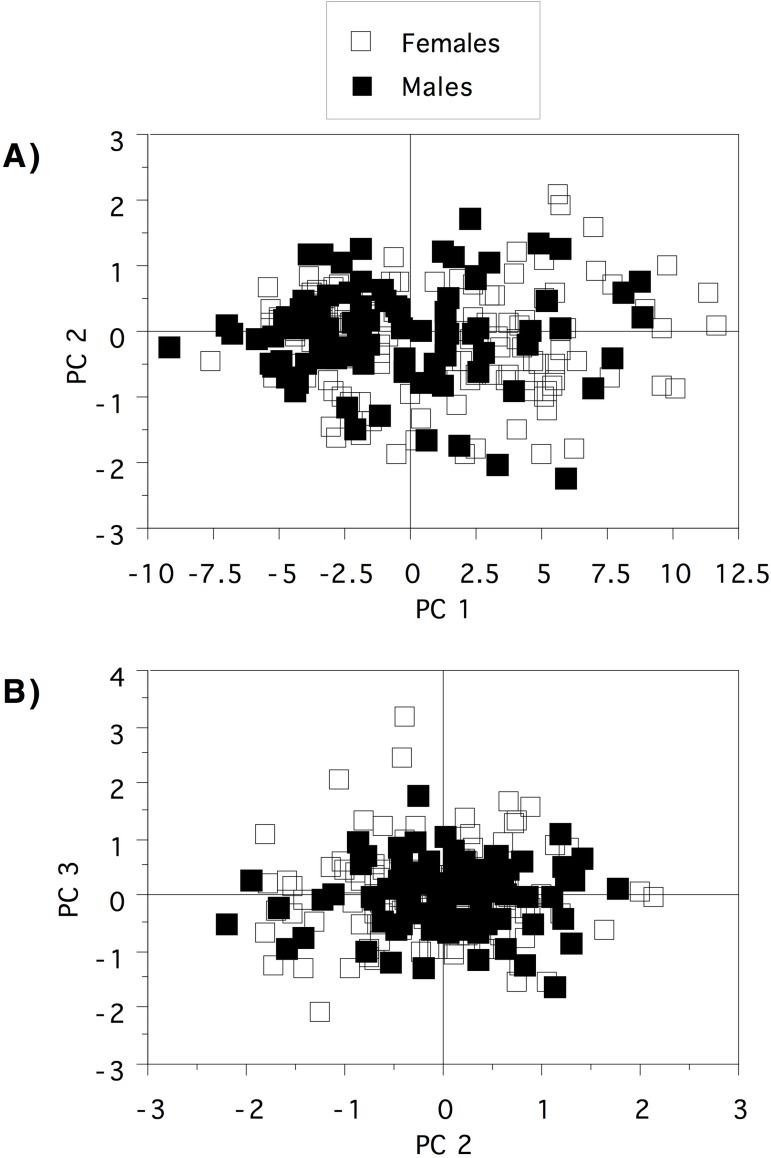
**Plots of the first and second (A) and second and third (B) Principle Components based on analysis of the morphological data set (see text for details) to show differences between the sexes**.

PCA was performed on the entire data set of 21 characters with imputation of missing values. Together, the first three factors explain 88.24% of the variation (PC1 = 82.47%, PC2 = 3.09%, PC3 = 2.67%), with PC1 interpreted as primarily accounting for body size differences, summarised in Fig 4. The plot of PC1 and PC2 (Fig 4A), and PC2 and PC3 (Fig 4B) clearly shows no morphological differentiation between the three haplotype groups. ANCOVA analyses of sexual size dimorphism in individual characters showed that, at the same SVL, females tend to be slightly larger than males in some variables, but these differences were subtle and there were few differences between males and females when other covariates were used (S1 Table). Replotting PCA values by sex rather than genetic group demonstrates that males and females are morphologically similar (Fig 5).

### Reproductive data and analysis

Specimens collected from the Geraldton region were too few to allow robust comparison with other regions, but observations of a female collected in October with tiny follicles but mature oviducts suggested that breeding had just occurred. Mean GSI for males (N = 4) and females (N = 3) collected in September and October were 6.6 and 8.3 respectively. The larger samples from the Swan Coastal Plain/northeastern and southeastern regions spanned months where frogs were likely to have been captured while foraging on the surface (March to August) or within breeding choruses from spring to early summer (Sept to December). The ad-hoc nature of these collections and their broad spatial extent precluded any statistical analysis of temporal trends in reproductive state, but visual inspection of the GSI by month showed that GSI increased in both sexes from January-December (slopes all positive). The increase was more marked for females from the Swan Coastal Plain/northeastern region, which had high GSI from October to December (mean 11.2, range 1.1–19.1, N = 54). In contrast, females from the southeastern clade with high GSI (>9, with follicles sizes ~5.0 mm) were collected from March−May and in September, November and December. A single female with tiny follicles and a mature oviduct (WAMR103745) collected at Jerramungup at the end of January provided the only conclusive evidence for recent breeding in this clade. Across the entire female dataset, female GSI differed significantly between these two clades (two tailed t-test, p = <0.0001), with the average GSI of females from the Swan Coastal Plain/northeastern clade being 9.9 (range 1.0–19.1, N = 68) relative to 5.7 (range 1.1–11.6, N = 35), for the southeastern females.

The increasing GSI of male specimens from January to December suggested peak reproductive activity in late spring and early summer, which is consistent with observed peaks in *M*. *gouldii* choruses in habitats on the Swan Coastal Plain but not with the timing of egg deposition which does not occur until February near Perth [27]. GSI was significantly greater for Swan Coastal Plain/northeastern males relative to southeastern males (two tailed t-test, p = 0.017), averaging 6.6 (range 3.7–9.4, N = 35) and 5.7 (range 2.7–10.5, N = 35) respectively.

### Call data

Calls of *Arenophryne rotunda* and *Myobatrachus gouldii* were described by Roberts [27, 31]. Fig 3 shows exemplars from the sets of calls analysed in those two papers. The second call of *M*. *gouldii* is from a site approximately 42 km NNE of the site reported by Roberts [27]. Across its range the calls of *M*. *gouldii* are structurally very similar with a slow, symmetric rise then decline in call amplitude, no evidence of frequency modulation but some evidence of two frequency components (sound spectrograms—Fig 3). Qualitative assessment of calls across the species distribution suggests there is no evidence of call differentiation across the range of this species (JD Roberts, personal observation has heard frogs calling immediately north and south of the Stirling Ranges and in the eastern and southeastern wheatbelt near Borden and Gnowangerup in spring consistent with frogs near Perth, but at Mount Hampton and Mt Walker calls were heard in mid-winter). Calls of *Arenophryne rotunda* and *Metacrinia nichollsi* reach peak amplitude by the second pulse in the call, then either maintain amplitude (*Metacrinia*) or, decline across the call duration (*Arenophryne*) with an acceleration of pulse rate evident at the end of the call in both species. Despite these differences, the calls of all three species sound very similar when allowance is made for body size differences and the strong negative correlations of dominant frequency and body size differences in Australian frogs [49].

## Discussion

We have produced the first phylogeny for populations of the turtle frog, *M*. *gouldii*, across its range, and present this genetic information alongside analyses of morphological and reproductive variation, to shed light on the evolutionary history of this bizarre species.

### Phylogeny and biogeography of *M*. *gouldii* populations

Our phylogenetic analyses revealed low genetic diversity within *M*. *gouldii* (Fig 2, S2 and S3 Figs). The concatenated RAxML analysis revealed a number of shallow clades or clusters but with little support for intraspecific relationships. However, this variation is broadly geographically structured, and taken together with the haplotype network, we identified five groups that correspond to discrete geographic regions within the range of *M*. *gouldii* (Fig 2). The Swan Coastal Plain (SCP) includes remnant *Banksia* woodlands across the greater Perth metropolitan area, and the group is non-monophyletic in our ML tree (S3 Fig). Northeastern (NE) is comprised of a divergent cluster of samples about 250km inland northeast of Perth. Geraldton (G) consists of samples near the Geraldton coast, and a population from ~35km east of Geraldton. Finally, southeastern (SE) contains samples from across the wheatbelt, east of the Darling Scarp. This haplotype group covers the widest portion of *M*. *gouldii*’s range yet displays very little mtDNA variation (0.37% maximum uncorrected genetic distance in ND2). A single individual from near Wyalkatchem is divergent from all other groups. Together these five groups are characterised by a maximum genetic distances of 3.3% based on ND2. Despite shallow structure, there are no shared haplotypes between regions (Fig 2).

Glacial arid cycles of the Plio-Pleistocene have been pivotal to generating species diversity in SWA, with many studies identifying these climatic fluctuations as major drivers of inter and intra-specific variation in flora and fauna [5, 13, 16, 37, 38]. At the same time, glacial cycles also are thought to have extinguished diversity in a number of taxa occupying regions that experienced particularly hostile arid conditions, enhanced by low-lying homogeneous topography and increased distance from the coast [50]. Centres of ancestral diversity are often characterised by high haplotype diversity, containing diversity not removed through founder effects or population bottlenecks [51]. The clades that harbour the most diversity within *M*. *gouldii* are largely coastal (SCP, NE and G), and it is likely that these regions remained climatically hospitable, allowing lineages to persist and to some degree diversify, during arid glacial cycles [37, 52]. In contrast, the SE group has a wide distribution including much of inland SWA and has much lower genetic diversity. This could indicate a severe population bottleneck as a result of increased aridity in these inland areas followed by wide-scale dispersal. Alternatively, as *M*. *gouldii* spends the vast majority of the year underground in leached sandy soils, soil and associated floristic characteristics are likely to be pivotal in dictating the species’ distribution. Recent arid cycles of the Quaternary were associated with a loss of rainforest vegetation and a gradual increase in sand coverage [4, 12], and may have enabled a rapid radiation of the SE group into a region that previously was unsuitable for *M*. *gouldii*.

There is some consistency between the phylogeographic patterns we have identified in *M*. *gouldii* and other SWA taxa. The Swan Coastal plain is composed of three major, recent, sedimentary systems—Bassendean, Spearwood and Quindalup, generally of Holocene or Pleistocene age [53]. The eastern boundary of the Swan Coastal Plain is marked by the Darling Scarp, a low-lying ridge with a maximum height of 582m (Mt Cooke) but an average height closer to 300m. The Darling Scarp is thought to have its origins in the Cretaceous [54], has been identified as a barrier to gene flow in a number of taxa [13, 14, 55], and marks the edge of many species’ ranges (e.g. plants [12]; frogs [56]). The Darling Scarp and the associated forest systems (jarrah, and further south karri and tingle: the High Rainfall Province of Hopper & Gioia [38]) separate the SCP and SE clades. The escarpment has a higher rainfall than the coastal plain or the wheatbelt, and is generally devoid of sandplain so is not suitable habitat for *M*. *gouldii* [27]. However, there is one recently collected sample from sand patches near the Blackwood River at Bridgetown (WAM R116034) in what is otherwise jarrah forest. There is also a clear division between the SCP clade and the NE clade, consistent with variation in *Acacia* species [16].

There is no deep subdivision across the range of *Myobatrachus*, consistent with data for *Heleioporus* species with similar ranges [57, 58] and limited data for *Neobatrachus* species in the Western Australian wheatbelt [59, 60], and also with some similarities to several other groups where one species has spread into the wheatbelt but not differentiated from a cluster of related species in the Swan Coastal Plain or southwest forest regions (Figure 9 Model 3 of [8]).

### Comparison of *M*. *gouldii* to *Arenophryne* and *Metacrinia*

The shallow phylogeographic structure in the mtDNA locus, with a maximum uncorrected genetic distance of only 3.3% between individuals, is consistent with, or lower than, intraspecific divergences found in studies of many other frogs [40, 61–63]. For comparison, pairwise genetic distances between the two *Arenophryne* species ranged from 5.5% to 6.4% [17] and between the three *M*. *nichollsi* mtDNA clades from 2.76% to 5.42% [37] Three of the coding nDNA genes we sequenced were almost completely invariant and any differences were not associated with geographic location. POMC is commonly used for species-level studies on frogs [42, 43, 64, 65] as is BDNF [41, 44, 65]. The intron RPL3int5, displayed greater variation, but with a high degree of incomplete lineage sorting and with little phylogeographic structure (S2 Fig).

Morphological characters such as those examined in this study were found to differ consistently between *A*. *rotunda* and *A*. *xiphorhyncha*, (also fossorial, psammophilic species), and this was thought to reflect differences in sand type [23]. As *M*. *gouldii* is widely distributed and also specialised to sandy soils, adaptations to subtle environmental differences might also be expected. However, our morphological data revealed no significant differences in morphological characters between genetic clades associated with distinct geographic regions (Fig 4). Unfortunately, due to low sampling from Wicherina and Spalding Park, these two genetically and geographically isolated clades could not be analysed individually in the morphological analyses.

Reproductive traits did show some divergence between the SCP/NE and SE clades, with larger follicle sizes relative to body size being characteristic of SCP/NE females. The higher GSI in the coastal clade most likely relates to the slightly larger follicles produced by relatively smaller females (data not presented). Assuming equal investment in reproduction across populations, females in the SCP/NE are likely to deposit smaller clutches of larger eggs relative to females from inland SE. No empirical evidence is available to test this assertion, and indeed the only published reports of clutch sizes in *M*. *gouldii* range between 5–50 mature eggs from SCP populations [27, 30, 33]. A further difference in the reproductive data was the retention of large follicles in females from the SE clade throughout the year, while follicle sizes tended to increase from January to December in the SCP/NE group, as plotted by Watson and Saunders [30]. This suggests greater seasonality or synchronicity of breeding in coastal clades, and more opportunistic breeding in inland clades, which should be adaptive in arid environments with unpredictable rainfall. The two observations of winter calling in eastern wheatbelt populations fits with this suggestion. Data on the timing of breeding choruses across the two clades could allow a test of this hypothesis, but given that *M*. *gouldii* pairs breed deep underground and can delay mating after courtship by several months [27, 34] even signals as obvious as breeding choruses may not fully reveal the seasonal patterns of reproduction in each clade.

## Supporting information

S1 Fig Map depicting locations of *Myobatrachus gouldii* specimens sampled for sequence data and morphology. Sampling gaps can be seen by comparing the ‘Morphology’ and ‘Genotyped’ markers to Western Australian Museum (WAM) locality data, from which the range of *M*. *gouldii* is inferred.
(DOC)

S2 Fig Representative parsimony phylograms for each of the four nDNA genes sequenced in this study. For each gene one of the 1,000 saved parsimony trees is shown. Branch lengths are indicated for each gene. *Myobatrachus gouldii* samples are indicated by a black line and other taxa represent outgroup species.
(PDF)

S3 Fig Phylogeny of 42 *Myobatrachus gouldii* samples and outgroups based on the combined nd2 and rpl3 data, and their distribution across eastern Australia. Here we show the relationships among the ve clades based on a concatenated RAxML analysis. Numbers beside nodes refer to ML bootstrap support.
(PDF)

S1 Text PCR and Sequencing methods (DOCX)

S2 Text Voucher numbers from the Western Australian Museum (prefix WAMR removed) of *Myobatrachus gouldii* specimens used in the morphometric data set. (DOCX)

S1 Table Summary of Analysis of Covariance (ANCOVA) for sexual size dimorphism. Variables of interest were examined against a stable co-variate as noted. Homogeneity of slopes tests were conducted initially. If the slopes were homogeneous an intercepts test was done to test for dimorphism. The interpretation of each test is shown.
(DOC)
