# Supplementary material for: Evolutionary and natural history of the turtle frog, Myobatrachus gouldii, a bizarre myobatrachid frog in the southwestern Australian biodiversity hotspot
Source: PLoS One. 2017 Mar 15;12(3):e0173348. doi: 10.1371/journal.pone.0173348 (PMC5351994; doi:10.1371/journal.pone.0173348)

**S1 Fig**. **Map depicting locations of *Myobatrachus gouldii* specimens sampled for sequence data and morphology.** Sampling gaps can be seen by comparing the ‘Morphology’ and ‘Genotyped’ markers to Western Australian Museum (WAM) locality data, from which the range of *M. gouldii* is inferred.


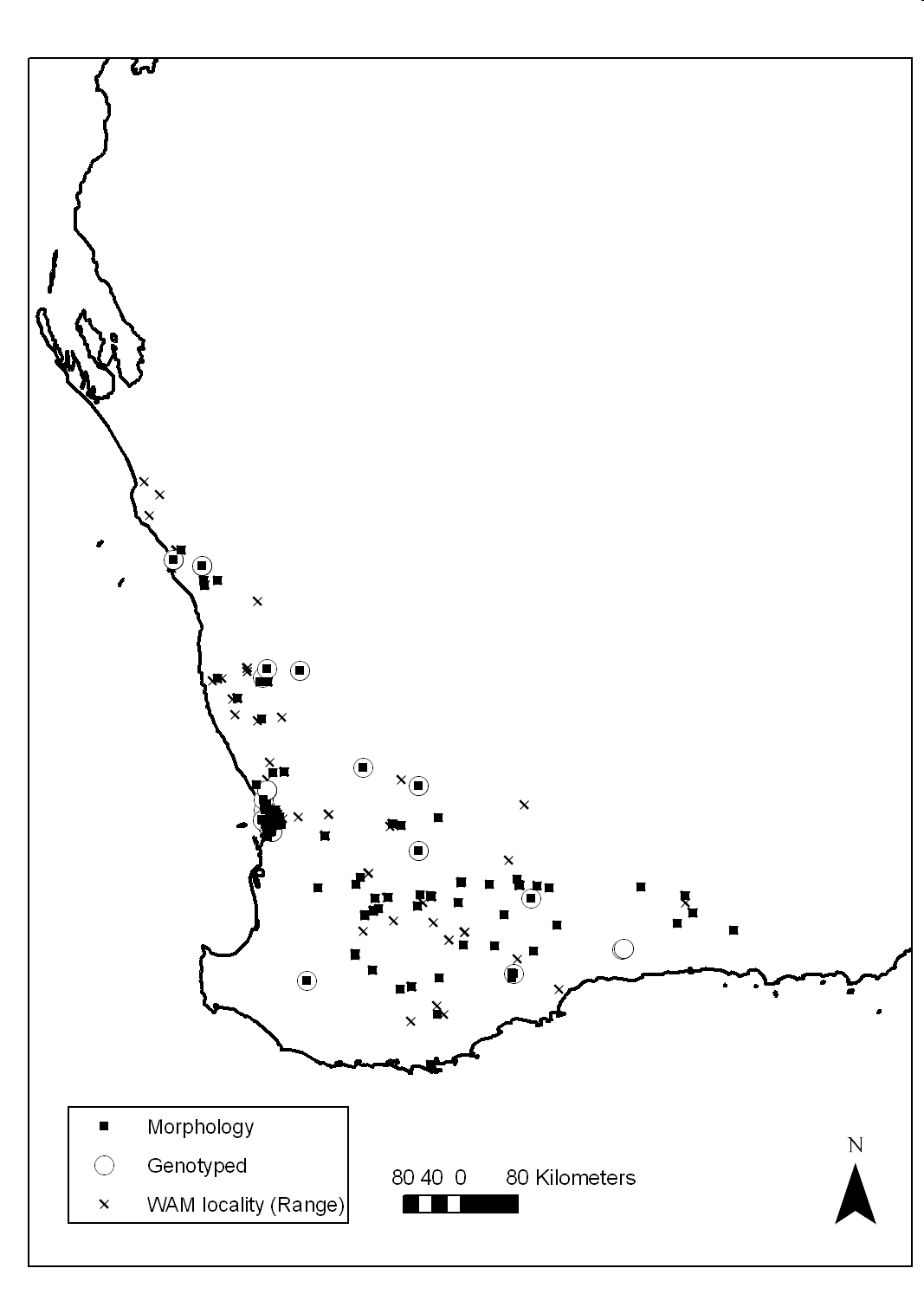

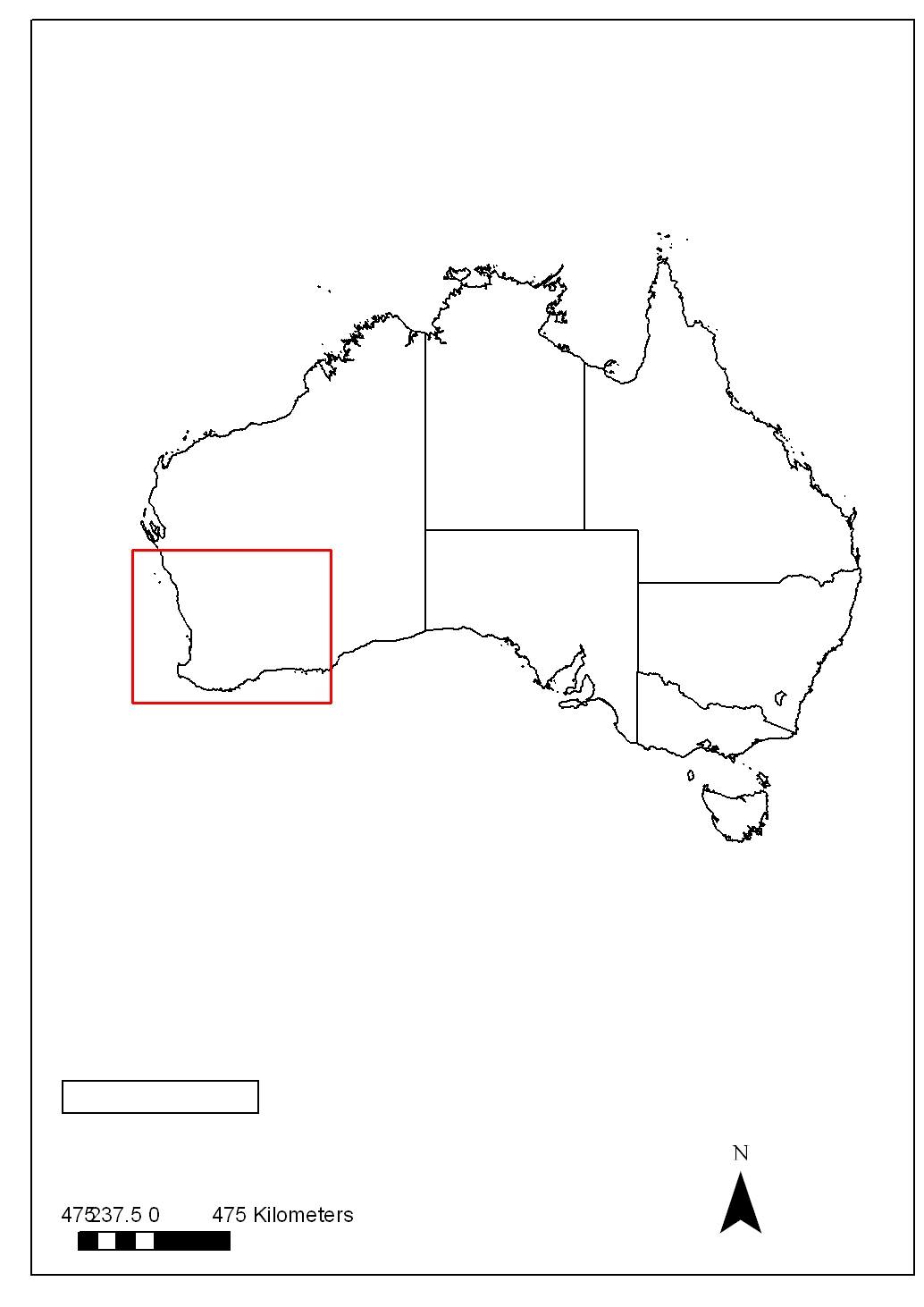

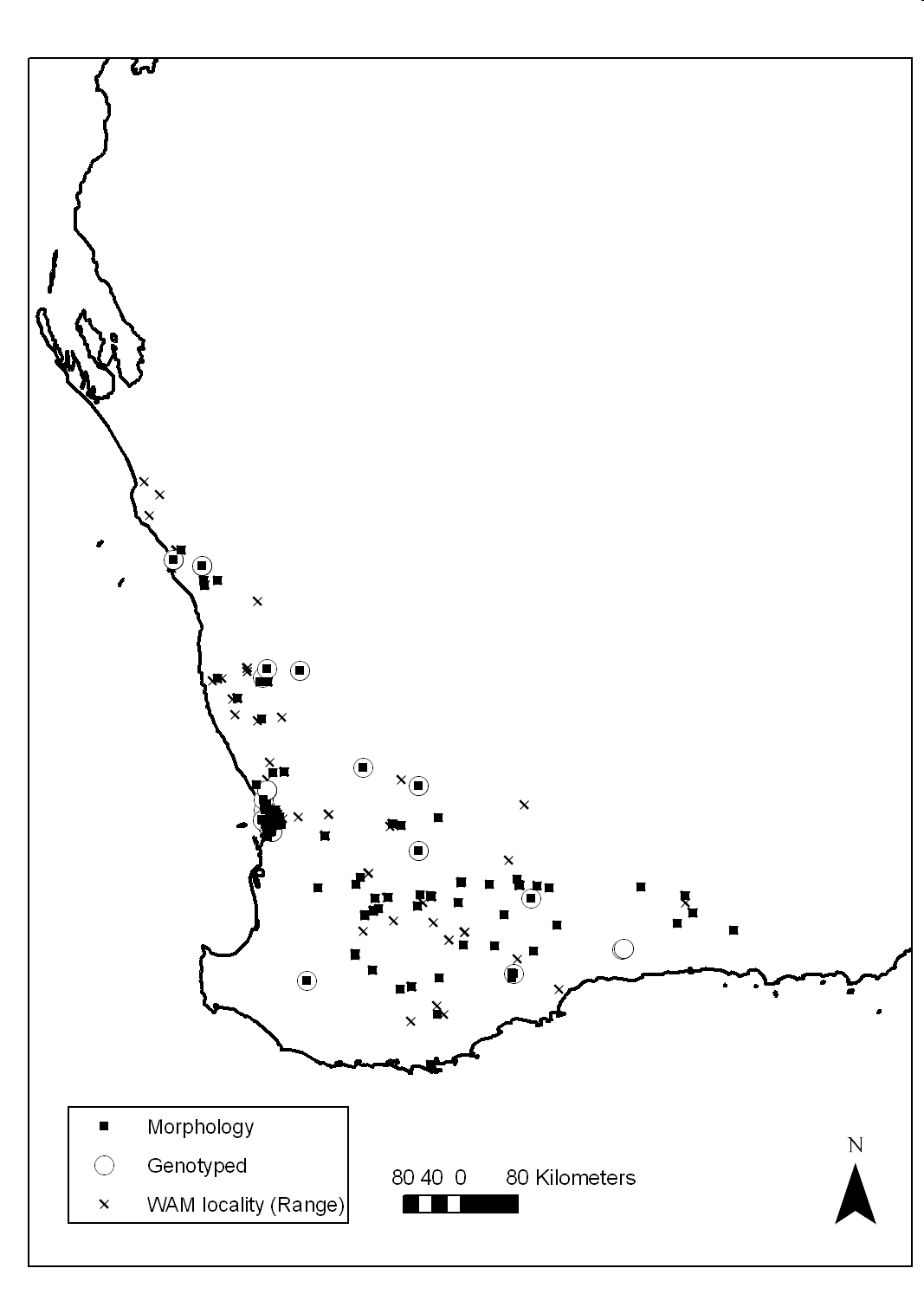

Supplement: S1 Fig — Sampling gaps can be seen by comparing the ‘Morphology’ and ‘Genotyped’ markers to Western Australian Museum (WAM) locality data, from which the range of M. gouldii is inferred. (DOC) [file pone.0173348.s001.doc]
