# Supplementary figures and images for: Evolutionary and natural history of the turtle frog, Myobatrachus gouldii, a bizarre myobatrachid frog in the southwestern Australian biodiversity hotspot
Source: PLoS One. 2017 Mar 15;12(3):e0173348. doi: 10.1371/journal.pone.0173348 (PMC5351994; doi:10.1371/journal.pone.0173348)

## BNDF2

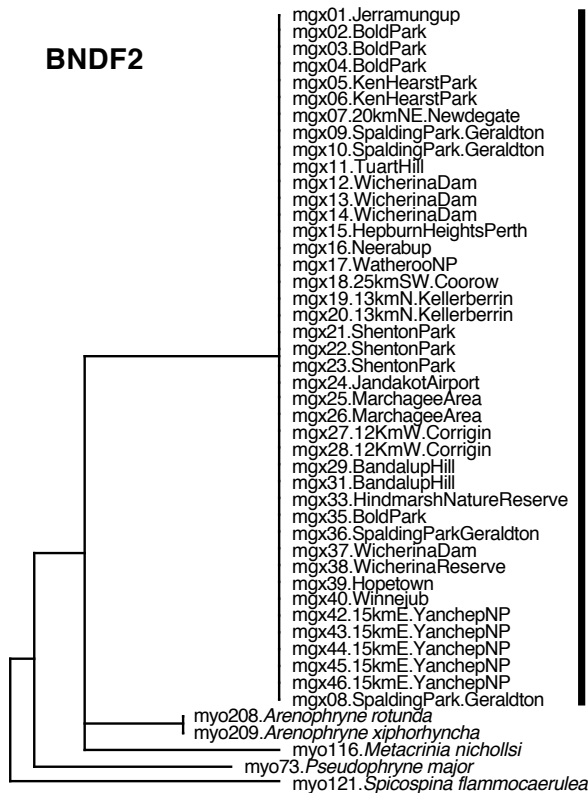

— 0.5 changes

## NTF32

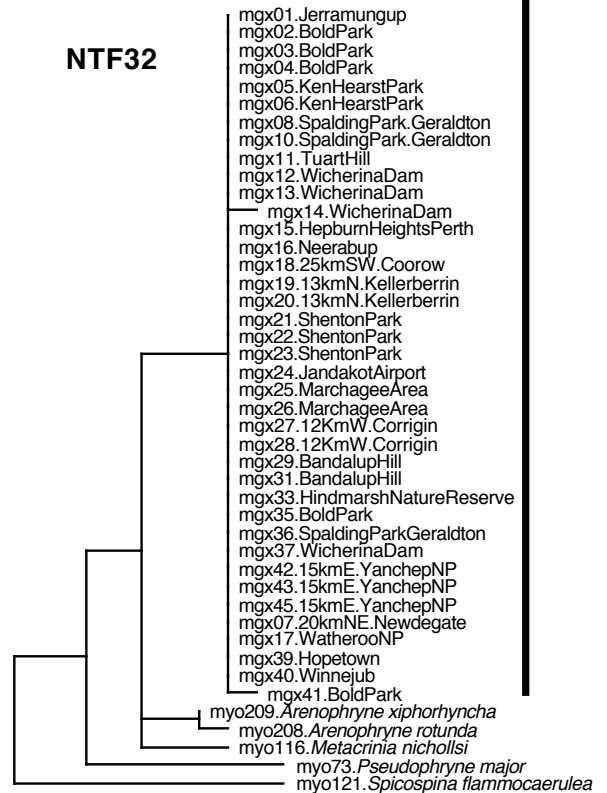

— 0.5 changes

## POMC

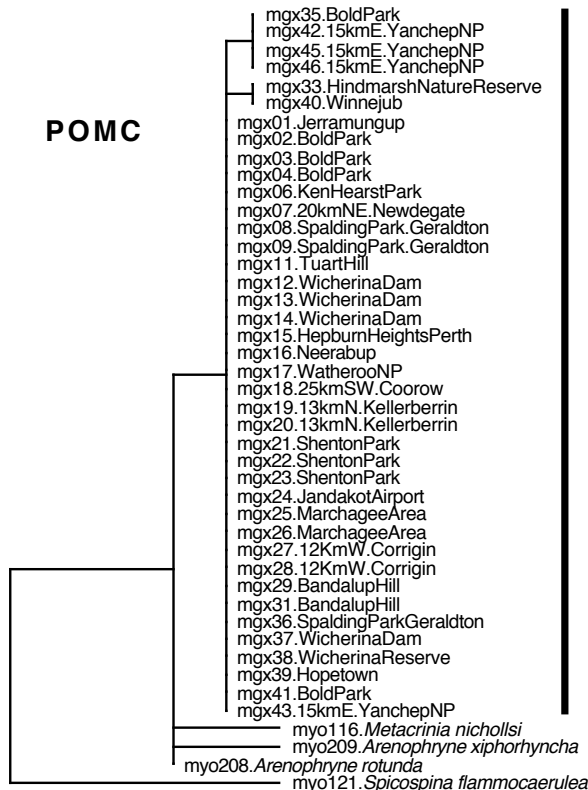

— 0.5 changes

## RPL3intron

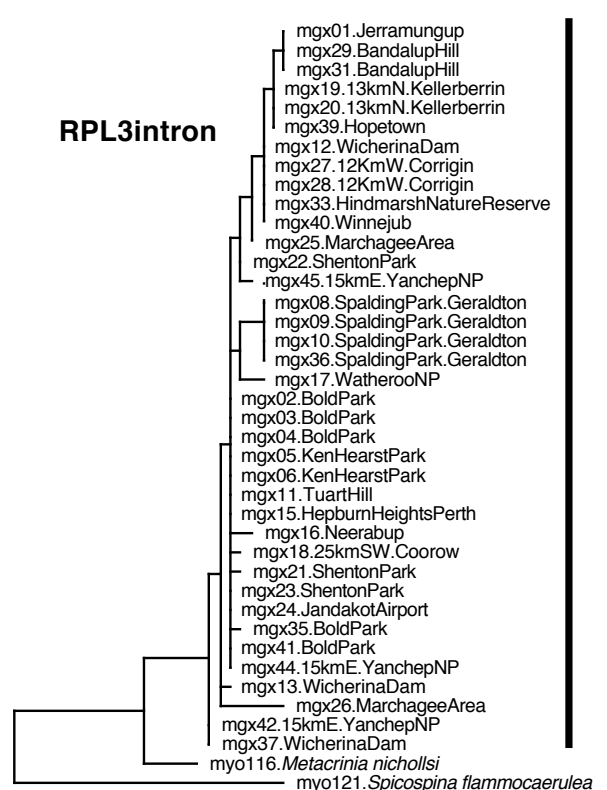

— 1 change

Supplement: S2 Fig — For each gene one of the 1,000 saved parsimony trees is shown. Branch lengths are indicated for each gene. Myobatrachus gouldii samples are indicated by a black line and other taxa represent outgroup species. (PDF) [file pone.0173348.s002.pdf]

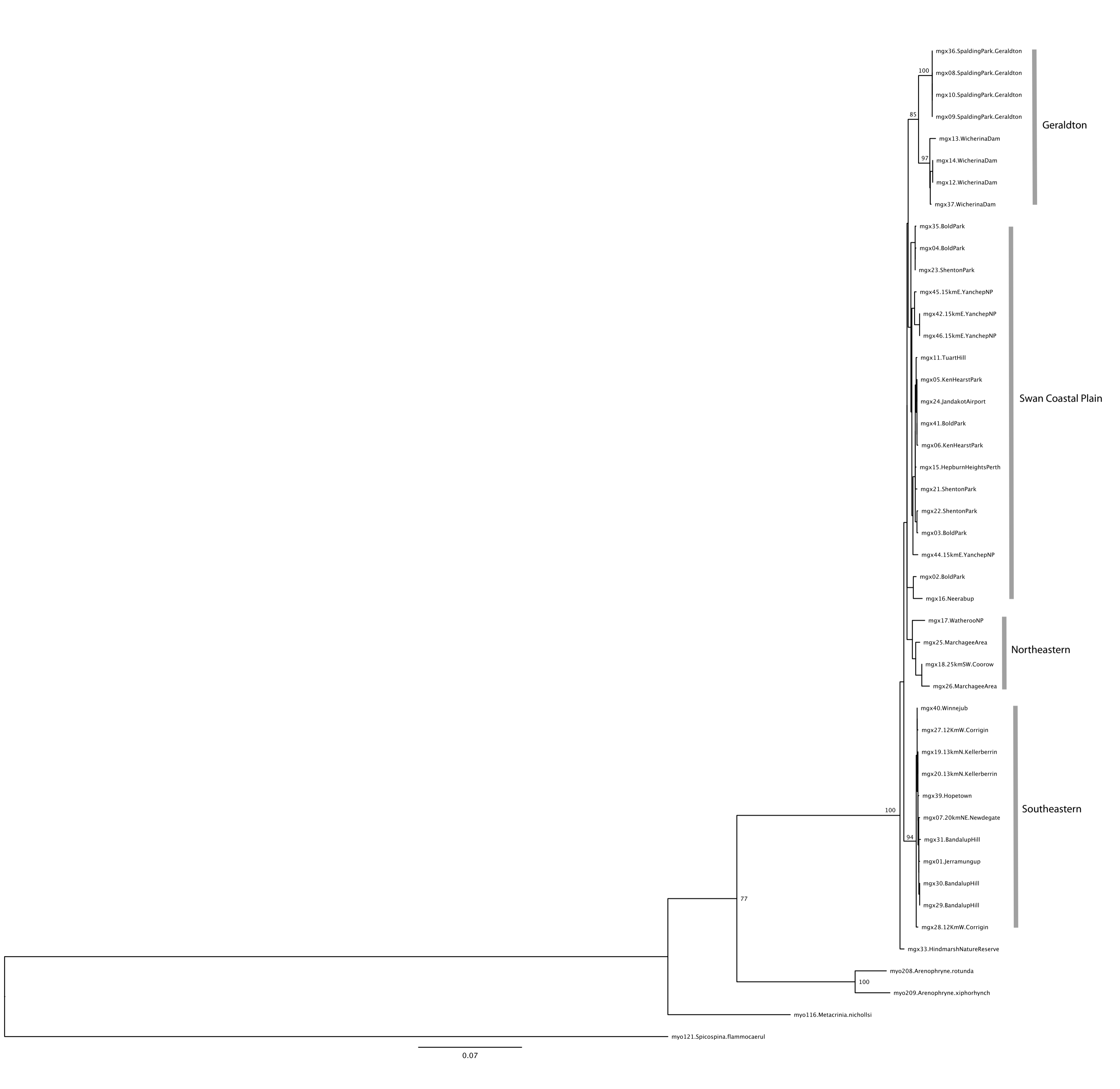

Supplement: S3 Fig — Here we show the relationships among the ve clades based on a concatenated RAxML analysis. Numbers beside nodes refer to ML bootstrap support. (PDF) [file pone.0173348.s003.pdf]
