# Supplementary material for: Evolutionary and natural history of the turtle frog, Myobatrachus gouldii, a bizarre myobatrachid frog in the southwestern Australian biodiversity hotspot
Source: PLoS One. 2017 Mar 15;12(3):e0173348. doi: 10.1371/journal.pone.0173348 (PMC5351994; doi:10.1371/journal.pone.0173348)
Supplement: S1 Text — (DOCX) [file pone.0173348.s004.docx]

PCR and Sequencing methods

A modified CTAB method was used to extract DNA which was then suspended in TE buffer and stored at -20°C. PCR amplification was performed using a Corbett PC-960C cooled thermal cycler and negative controls were run for all amplifications. Amplification was performed in a 25μl reaction mix consisting of 2.5μl 10xPCR Buffer, 1.5μl 50mM MgCl_2_, 1.25μl dNTPs (10mM), 1.0μl of both forward and reverse primers (10pmol), 17.75μl double distilled water, 0.1μl Platinum Taq (5 units/μl) and 1μl of template DNA. The primers used are detailed in Table 2. Amplification of the mitochondrial DNA fragments was conducted with an activation step of 94°C for 4 mins, touchdown from 94°C (30s), 70°C (20s) and 72°C (1m30s) to 94°C, 45°C, 72°C (annealing temperature down 5°C every 2 cycles), followed by 35 cycles of 94°C (30s), 40°C (30s) and 72°C (45s), with a final extension of 72°C for 4 mins. Amplification of nuclear genomic DNA was conducted using an activation step of 95°C for 4mins, touchdown from 95°C for 15s, 55°C for 15s and 72°C for 30s, to 91°C, 51°C and 72°C (annealing temperature down 1°C each 2 cycles), followed by 30 cycles of 90°C (15s), 50°C (15s) and 72°C (30s) with a final extension of 72°C for 4mins. For RPL3int5: activation step of 95°C for 4 mins, touchdown from 95°C for 30s, 55°C for 30s and 72°C for 1min, to 95°C (30s), 45°C (30s) and 72°C (1min) (annealing temperature down 5°C every 2 cycles), followed by 20 cycles of 95°C, 50°C and 72°C, and 30 cycles of 95°C, 47°C and 72°C, with a final extension of 72°C for 6mins. Approximate concentration of amplification products was determined after each extraction and amplification step by electrophoresis in a 1.5% agarose gel stained with 6.0μl ethidium bromide and visualized under ultra-violet light. PCR products were gel purified using the ExoSap cleanup method (nuclear markers), or ammonium acetate precipitation (ND2). Cycle-sequencing reactions were performed in reaction volumes of 20μl, consisting of 0.7μl (nuclear markers) or 1.0μl (ND2) BigDye (Applied Biosystems), 4.5μl 5x Sequencing Buffer, 0.32μl Primer (10 pmol/μl), 13.5μl (nuclear) or 13.2μl (ND2) doubly distilled water, and 1.0μl purified PCR product. PCR sequencing cycles were performed using a Corbett PC-960C cooled thermal cycler, with a denaturation step at 94^°^C for 5s, annealing at 50^°^C for 10s, and extension at 60^°^C for 4 minutes, for 25 cycles. To precipitate sequence products and to remove all unincorporated nucleotides, a mixture of cold 40μl of 95% Ethanol and 3μl Sodium Acetate (3M, ph: 4.6-5.2) was added to each sample and left for 15 minutes at room temperature. Precipitated DNA was pelleted and washed thrice in 150μl of 70% Ethanol. Pellets were dried before being dissolved in 20μl of HiDi formamide and run on an ABI 3100 auto-sequencer.
