# Supplementary material for: Evolutionary and natural history of the turtle frog, Myobatrachus gouldii, a bizarre myobatrachid frog in the southwestern Australian biodiversity hotspot
Source: PLoS One. 2017 Mar 15;12(3):e0173348. doi: 10.1371/journal.pone.0173348 (PMC5351994; doi:10.1371/journal.pone.0173348)
Supplement: S2 Text — (DOCX) [file pone.0173348.s005.docx]

**S2 Text.** **Voucher numbers from the Western Australian Museum (prefix WAMR removed) of *Myobatrachus gouldii* specimens used in the morphometric data set.**

39, 580, 966, 1334, 1348, 1528, 1674, 1778, 2263, 2265, 2381, 2590, 2801, 3874, 4626, 4919, 4937, 5898, 6937, 6996, 7814, 7823, 7902, 8175, 8372, 8464, 9566, 10139, 10736, 10963, 11613, 12847, 13932, 19816, 19997, 22600, 25369, 26734, 33370, 33371, 3440, 42963, 42971, 43997, 44451, 46127, 46549, 49795, 49796, 49797, 51589, 51590, 5159, 51592, 51593, 51594, 51595, 52494, 52495, 53160, 57023, 58108, 58109, 58323, 5882, 60817, 60818, 60821, 60823, 60824, 60825, 61792, 61793, 61795, 61796, 61797, 61799, 61800, 61801, 61802, 61803, 61805, 61806, 61807, 62565, 62566, 62567, 62568, 62569, 62571, 68481, 68485, 68486, 68497, 68502, 68503, 68513, 68514, 68516, 68517, 68518, 68519, 68521, 68522, 68525, 68526, 68528, 68529, 68531, 68537, 68539, 68540, 68541, 68543, 68544, 68546, 68548, 68551, 68554, 68555, 68557, 68559, 68562, 68564, 69960, 73913, 73914, 78870, 78871, 78873, 81404, 84161, 88132, 88133, 88368, 88369, 88370, 89449, 90933, 93034, 93058, 93705, 94458, 94465, 94492, 95449, 95450, 101057, 103745, 108429, 115075, 115076, 115077, 115103, 115975, 116034, 116074, 116076, 116077, 116415, 118091, 118124, 118335, 120084, 120300, 120305, 120306, 127623, 130123, 130126, 130134, 130136, 130234, 130465, 130473, 130897, 130898, 130899, 130915, 130917, 130981, 130987, 131511, 131512, 131513, 131515, 131516, 131520, 133461, 133462, 133484, 133486, 133487, 133544, 133672, 134440, 134449, 134450, 134477, 136494, 141705, 142739, 143375, 143714, 143767, 143831, 143897, 144078, 144079, 144139, 144140, 144894, 144895, 144984, 146470, 146471, 149210, 149224, 149510, 149516, 149520, 149541, 149565, 149582, 149583, 149596, 149610, 149640, 149883, 149928, 150209, 150222, 154371, 154372.
