# Supplementary material for: Evolutionary and natural history of the turtle frog, Myobatrachus gouldii, a bizarre myobatrachid frog in the southwestern Australian biodiversity hotspot
Source: PLoS One. 2017 Mar 15;12(3):e0173348. doi: 10.1371/journal.pone.0173348 (PMC5351994; doi:10.1371/journal.pone.0173348)
Supplement: S1 Table — Variables of interest were examined against a stable co-variate as noted. Homogeneity of slopes tests were conducted initially. If the slopes were homogeneous an intercepts test was done to test for dimorphism. The interpretation of each test is shown. (DOC) [file pone.0173348.s006.doc]

**S1 Table. Summary of Analysis of Covariance (ANCOVA) for sexual size dimorphism.** Variables of interest were examined against a stable co-variate as noted. Homogeneity of slopes tests were conducted initially. If the slopes were homogeneous an intercepts test was done to test for dimorphism. The interpretation of each test is shown.

|  |  | **Homogeneity of slopes test** | | | **Intercepts** | | | | |  |
| --- | --- | --- | --- | --- | --- | --- | --- | --- | --- | --- |
| Covariate | Variable of interest | Fdf | F | P | Fdf | F | | P | | Result |
| SVL | IIL | 1,194 | 9.767 | 0.0021 |  |  | |  | |  |
|  | Hand Length | 1,194 | 1.917 | 0.1678 | 1,195 | 13.897 | | 0.0003 | | females longer |
|  | Foot Length | 1,187 | 3.797 | 0.0528 | 1,188 | 7.0601 | | 0.0086 | | females longer |
|  | Tarsotibular Length | 1,192 | 6.334 | 0.0127 |  |  | |  | |  |
|  | Head Length (Tymp to Nose) | 1,181 | 2.275 | 0.1332 | 1,182 | 6.988 | | 0.0089 | | females longer |
|  | Head Length (Jaw to Nose) | 1,158 | 4.891 | 0.0284 |  |  | |  | |  |
|  | Head Width | 1,166 | 0.05 | 0.8233 | 1,167 | 4.75 | | 0.0307 | | females wider |
|  | Mouth Width | 1,130 | 4.218 | 0.042 |  |  | |  | |  |
|  | Shoulder Width | 1,191 | 2.197 | 0.1399 | 1,192 | 7.351 | | 0.0073 | | females wider |
|  | Tympanum Width | 1,130 | 1.688 | 0.1961 | 1,131 | 0.014 | | 0.9056 | | no difference |
|  | Arm Width | 1,190 | 0.029 | 0.864 | 1,191 | 0.123 | | 0.7258 | | no difference |
|  | Long Toe Length | 1,192 | 1.5434 | 0.217 | 1,193 | 12.362 | | 0.0005 | | females longer |
|  | Inner Toe Length | 1,192 | 1.163 | 0.2823 | 1,193 | 149.038 | | 0.0001 | | females longer |
|  | Finger Length | 1,195 | 3.357 | 0.0684 | 1,193 | 5.0681 | | 0.0255 | | females longer |
|  | Thumb Length | 1,195 | 1.835 | 0.1771 | 1,193 | 2.246 | | 0.1356 | | no difference |
|  | Wrist Width | 1,195 | 5.81 | 0.0169 |  |  | |  | |  |
|  | Eye Naris Distance | 1,193 | 7.767 | 0.0169 |  |  | |  | |  |
|  | Inter Orbital Span | 1,194 | 1.886 | 0.1712 | 1,195 | 6.846 | | 0.0096 | | females wider |
|  | Inter Narial Span | 1,194 | 4.489 | 0.0354 |  |  | |  | |  |
|  | Eye Length | 1,194 | 0.059 | 0.6903 | 1,195 | 0.015 | | 0.9035 | | no difference |
|  |  |  |  |  |  |  | |  | |  |
| Hand Length | Finger Length | 1,218 | 0.137 | 0.7116 | 1,219 | 0.13 | | 0.7192 | | no difference |
|  | Thumb Length | 1,218 | 0.023 | 0.8784 | 1,219 | 0.115 | | 0.7352 | | no difference |
|  | Wrist Width | 1,218 | 1.281 | 0.259 | 1,219 | 0.011 | | 0.9169 | | no difference |
|  |  |  |  |  |  |  | |  | |  |
|  |  | **Homogeneity of slopes test** | | | **Intercepts** | | | | |  |
| Covariate | Variable of interest | Fdf | F | P | Fdf | | F | | P | Result |
| Foot Length | Long Toe Length | 1,209 | 0.03 | 0.8635 | 1,210 | | 2.658 | | 0.1045 | females longer |
|  | Inner Toe Length | 1,210 | 3.304 | 0.0705 | 1,211 | | 1.29 | | 0.2574 | females longer |
|  |  |  |  |  |  | |  | |  |  |
|  |  |  |  |  |  | |  | |  |  |
| Head Width | Eye Naris Distance | 1,189 | 1.686 | 0.1957 | 1,190 | | 2.923 | | 0.089 | no difference |
|  | Inter Orbital Span | 1,189 | 0.974 | 0.325 | 1,190 | | 0.552 | | 0.4586 | no difference |
|  | Inter Narial Span | 1,189 | 0.138 | 0.715 | 1,190 | | 0.758 | | 0.385 | no difference |
|  | Eye Length | 1,189 | 1.101 | 0.2954 | 1,190 | | 0.153 | | 0.6958 | no difference |
|  | Tympanum Width | 1,125 | 4.691 | 0.0322 |  | |  | |  |  |
|  | Mouth Width | 1,125 | 1.763 | 0.1867 | 1,126 | | 2.62 | | 0.108 | no difference |
|  | Shoulder Width | 1,185 | 0.404 | 0.526 | 1,186 | | 0.002 | | 0.9637 | no difference |
|  | Head Length (Tymp to Nose) | 1,178 | 0.206 | 0.6502 | 1,179 | | 0.548 | | 0.4602 | no difference |
|  | Head Length (Jaw to Nose) | 1,178 | 0.254 | 0.6149 | 1,179 | | 4.036 | | 0.046 | females longer |
